# Impact of Race on Hyperparathyroidism, Mineral Disarrays, Administered Vitamin D Mimetic, and Survival in Hemodialysis Patients

**DOI:** 10.1002/jbmr.177

**Published:** 2010-07-07

**Authors:** Kamyar Kalantar-Zadeh, Jessica E Miller, Csaba P Kovesdy, Rajnish Mehrotra, Lilia R Lukowsky, Elani Streja, Joni Ricks, Jennie Jing, Allen R Nissenson, Sander Greenland, Keith C Norris

**Affiliations:** 1 Harold Simmons Center for Chronic Disease Research and Epidemiology, Los Angeles Biomedical Research Institute at Harbor-UCLA Medical Center Torrance, CA, USA; 2 Division of Nephrology and Hypertension, Los Angeles Biomedical Research Institute at Harbor-UCLA Medical Center Torrance, CA, USA; 3 UCLA David Geffen School of Medicine, UCLA School of Public Health Los Angeles, CA, USA; 4 Department of Epidemiology, UCLA School of Public Health Los Angeles, CA, USA; 5 Division of Nephrology, Salem Veterans Administration Medical Center Salem, VA, USA; 6 DaVita Inc. El Segundo CA, USA; 7 Department of Medicine, Charles Drew University Los Angeles, CA, USA

**Keywords:** MINERALS, HYPOCALCEMIA, RACIAL DISPARITIES, MINERAL AND BONE DISORDERS, CHRONIC KIDNEY DISEASE, PARICALCITOL

## Abstract

Blacks have high rates of chronic kidney disease, are overrepresented among the US dialysis patients, have higher parathyroid hormone levels, but greater survival compared to nonblacks. We hypothesized that mineral and bone disorders (MBDs) have a bearing on survival advantages of black hemodialysis patients. In 139,328 thrice-weekly treated hemodialysis patients, including 32% blacks, in a large dialysis organization, where most laboratory values were measured monthly for up to 60 months (July 2001 to June 2006), we examined differences across races in measures of MBDs and survival predictabilities of these markers and administered the active vitamin D medication paricalcitol. Across each age increment, blacks had higher serum calcium and parathyroid hormone (PTH) levels and almost the same serum phosphorus and alkaline phosphatase levels and were more likely to receive injectable active vitamin D in the dialysis clinic, mostly paricalcitol, at higher doses than nonblacks. Racial differences existed in mortality predictabilities of different ranges of serum calcium, phosphorus, and PTH but not alkaline phosphatase. Blacks who received the highest dose of paricalcitol (>10 µg/week) had a demonstrable survival advantage over nonblacks (case-mix-adjusted death hazard ratio = 0.87, 95% confidence level 0.83–0.91) compared with those who received lower doses (<10 µg/week) or no active vitamin D. Hence, in black hemodialysis patients, hyperparathyroidism and hypercalcemia are more prevalent than in nonblacks, whereas hyperphosphatemia or hyperphosphatasemia are not. Survival advantages of blacks appear restricted to those receiving higher doses of active vitamin D. Examining the effect of MBD modulation on racial survival disparities of hemodialysis patients is warranted. © 2010 American Society for Bone and Mineral Research.

## Introduction

In the United States, chronic kidney disease (CKD) is common and associated with major racial disparities.(1–3) Approximately one-third of the 400,000 US dialysis patients are African Americans, even though they comprise 14% of the US general population.(4,5) The racial discrepancies of dialysis incidence have persisted over the past decades. The 2006 incident dialysis patient rates in the African-American population continued to be 3.6 times greater than among non-Hispanic whites.(4,5) Notwithstanding the public health implications of the CKD racial disparities, there are some encouraging facts pertaining to race. Whereas some two-thirds of all US dialysis patients die within 5 years of initiating dialysis therapy, a death rate currently worse than most cancers,(6) African American with end-stage renal disease (ESRD) have consistently greater survival over the past two decades than non-Hispanic whites, with an annual death rate of only 187 per 1000 patient-years at risk versus 207 per 1000 patient-years at risk for non-Hispanic whites.(4,5) The causes and consequences of these disparities remain largely unknown.

The spectrum of kidney bone diseases,(7,8) also known as the *mineral and bone disorders* (MBDs),(9,10) is not only common and highly correlated with morbidity and mortality in CKD patients, but it also is one of the disease conditions with distinct features and severity across races.(11–15) In particular, African-American CKD patients appear to have higher levels of parathyroid hormone (PTH) and are more likely to receive treatment for it.(14,15) A recent epidemiologic study in incident hemodialysis patients suggested that therapy with activate vitamin D agents may be a potential explanation for the racial differences in survival.(16) It is not clear whether different doses of these medications have a bearing on survival, in particular, among prevalent hemodialysis patients, who comprise most of the ESRD population in the United States.(4,5)

Understanding these issues is time-sensitive because the so-called bundling payment for dialysis patient care in the United States is imminent.(17–19) The inclusion of MBD-specific medications such as active vitamin D compounds within a bundled dialysis payment can have adverse unintended consequences if the proposed case-mix adjustment does not take into account race, which may be an important determinant of dosing requirements and patient survival. We therefore hypothesized that African-American hemodialysis patients are among the most susceptible populations for potentially deleterious impacts of the currently proposed bundling system, which does not consider race in adjusting payments. We examined the distribution of biochemical measures of CKD-MBD and pattern of active vitamin D treatment across races in a large (over 100,000 subjects) and contemporary (July 2001 to June 2006) cohort of hemodialysis patients from a large dialysis organization (DaVita, El Segundo, CA, USA) with monthly blood to quarterly blood tests and treatment record over the entire 5 years. We also hypothesized that African-American hemodialysis patients have distinct survival associations with surrogates of MBDs and that higher doses of active vitamin D may explain their survival advantages.

## Methods

### Patients

We extracted, refined, and examined data from all individuals with CKD stage 5 who underwent hemodialysis treatment from July 2001 to June 2006, that is, for 5 consecutive years, in one of the outpatient dialysis facilities of a US-based large dialysis organization (LDO), that is, DaVita (prior to its acquisition of the former Gambro dialysis facilities). The study was approved by the Institutional Review Committees of both the Los Angeles Biomedical Research Institute at Harbor-UCLA Medical Center and DaVita Clinical Research. Because of the large sample size, the anonymity of the patients, and the nonintrusive nature of the research, the requirement for a written consent form was exempted.

### Clinical and demographic measures

Creation of the cohort has been described previously.(20–26) To minimize measurement variability, all repeated measures for each patient during any given calendar quarter, that is, over a 13-week or 3-month interval, were averaged, and the quarterly means in each of the 20 calendar quarters were used in time-dependent analyses. In addition to quarterly laboratory values, quarterly averaged values for paricalcitol dose greater than 0 and posthemodialysis dry weight [to calculate averaged body mass index (BMI)] also were calculated. *Dialysis vintage* was defined as the duration of time between the first day of dialysis treatment and the first day that the patient entered the cohort. The first (baseline) study quarter for each patient was the calendar quarter in which patient's vintage was more than 45 days during at least half the time of that quarter.

### Laboratory measures

Blood samples were drawn using uniform techniques in all the DaVita dialysis clinics and were transported to the DaVita Laboratory in Deland, Florida, typically within 24 hours. All laboratory values were measured by automated and standardized methods in the DaVita Laboratory. Most laboratory values were measured monthly, including urea nitrogen, creatinine, albumin, calcium, phosphorus, bicarbonate, and total iron-binding capacity (TIBC). Serum ferritin and intact PTH were measured at least quarterly. Hemoglobin was measured at least monthly in essentially all patients and weekly to biweekly in most patients. Most blood samples were collected before dialysis, with the exception of the postdialysis serum urea nitrogen (SUN) that was obtained to calculate urea kinetics. The *Kt*/*V* (single pool) was calculated using urea kinetic modeling (UKM) equations, as described elsewhere.(20) Albumin-corrected calcium concentration was calculated by subtracting 0.8 mg/dL for each gram per deciliter of serum albumin below 4.0 g/dL.(27)

### In-center-administered active vitamin D agents

Patients who received any injectable active vitamin D agent (ie, calcitriol, doxercalciferol, and/or paricalcitol) in the dialysis facility during each given calendar quarter were identified, and the administered doses were extracted. Since over 90% of DaVita patients who received any active vitamin D between 2001 and 2006 received paricalcitol (Zemplar, Abbott Laboratories, Abbott Park, IL, USA), the dose of administered paricalcitol was calculated in micrograms per week (µg/week) for each calendar quarter over the entire 5 years of the cohort for every hemodialysis patient. Patients who received any dose of paricalcitol were divided into groups of average dose less than 10 µg/week and 10 µg/week or more. For this study, data on oral medications including calcimimetics or phosphorus binders were not available. The calcimimetic cinacalcet became available in the United States during the last few months of this 5-year cohort.(28,29) Hence the cohort is virtually a precalcimimetic cohort.

#### Statistical and epidemiologic methods

Age-stratified analyses across two mutually exclusive racial groups were performed to examine the role of race in the distribution of relevant clinical and laboratory variables independent of age. For survival analyses, we employed proportional hazards regression using quarterly averaged variables, as described earlier. Patients who were transplanted, switched to peritoneal dialysis, or left DaVita clinics were censored at the time of the event. Plots of log[–log(survival rate)] against log(survival time) were performed to check the proportionality assumption. For each analysis, two levels of multivariate adjustment models were examined:

1. Unadjusted (or minimally adjusted) models that included mortality and censorship data, the predicting variable, and the entry calendar quarter (Q1 through Q20).
2. Case-mix-adjusted models that included all the preceding plus age, gender, race/ethnicity (African Americans and other self-categorized blacks, non-Hispanic whites, Asians, Hispanics, and others), diabetes mellitus, categories of dialysis vintage (<6 months, 6 months to 2 years, 2 to 5 years, and 5 years or more), primary insurance (Medicare, Medicaid, private, and others), marital status (married, single, divorced, widowed, and other or unknown), the standardized mortality ratio of the dialysis clinic during entry quarter, residual renal function during the entry quarter, and *Kt*/*V* (single pool) to represents the administered dialysis dose.

We did not include laboratory measures as additional adjustors in most regression models. In our view, results from such excessive adjustments, which we usually refer to as the *malnutrition-inflammation-cachexia syndrome* (MICS) controlling, are likely overadjusted and may introduce new sources of bias owing to possible inclusion of intermediates in the causal pathways of the associations under study. On the other hand, results from the unadjusted models are likely to be profoundly confounded owing to omission of such inherent confounders as age and gender. We thus prefer to base inferences on the case-mix-adjusted models.

Unadjusted and case-mix-adjusted logistic regression analyses were used to calculate the odds ratio of receiving the highest paricalcitol dose (>10 µg/week) versus the lowest dose (>0 to <10 µg/week). Most analyses were carried out with SAS Version 9.1 (SAS Institute, Inc., Cary, NC, USA).

## Results

**Fig. 1:**
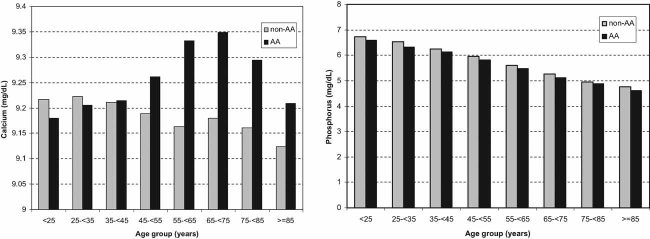
Comparing 3-month average serum calcium (*left panel*) and phosphorus (*right panel*) concentrations in the base calendar quarter across eight 10-year age increments in 139,328 hemodialysis patients including 43,974 African Americans (32%) and 95,354 non-African Americans (68%) from July 2001 to June 2001.

**Fig. 2:**
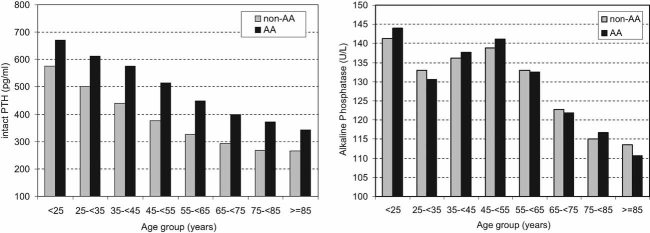
Comparing 3-month average serum intact PTH (*left panel*) and alkaline phosphatase (*right panel*) concentrations in the base calendar quarter across eight 10-year age increments in 139,328 hemodialysis patients including 43,974 African Americans (32%) and 95,354 non-African Americans (68%) from July 2001 to June 2001.

**Fig. 3:**
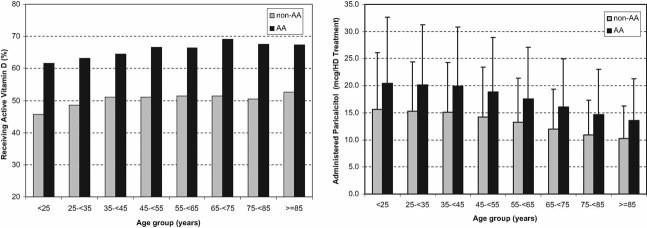
Pattern of active vitamin D administration in the examined calendar quarters across eight 10-year age increments in 139,328 hemodialysis patients from July 2001 to June 2001. (*Left panel*) Proportion of hemodialysis patients who received any dose of an active vitamin D compound (paricalcitol 58%, calcitriol 2%, doxercalciferol <1%, and no active vitamin D 38%) in 43,974 African-American and 95,354 non-African-American hemodialysis patients. (*Right panel*) The 3-month average administered paricalcitol dose (mean ± SD) among 49,674 hemodialysis patients (including 19,918 African Americans) who received any dose of paricalcitol during the calendar quarter.

The original 5-year (July 2001 to June 2006) national database of all DaVita hemodialysis patients included 152,058 adults. After deleting patients who did not maintain at least 45 days of hemodialysis treatment (9,151 patients from the first 19 calendar quarters and 3,579 patients from the last quarter), 139,328 hemodialysis patients remained, including 18% incident patients with a dialysis vintage of less than 6 months (see Supplemental Table S1 for category comparisons). The cohort included 43,974 African Americans (32%) and 95,354 non-African Americans (68%). Table 1 shows counts (in percentage) or means (± SD) of the relevant demographic, clinical, and laboratory variables in the entry calendar quarter of the patients across eight 10-year increments of age from younger than 25 years to older than 85 years of age. African Americans younger than 65 years of age and all non-African-American hemodialysis patients included more men than women, whereas this ratio reversed among elderly African Americans. Diabetes mellitus proportion was lower in younger African Americans than in non-African Americans, whereas this relationship reversed among older patients. Delivered *Kt*/*V* and serum concentrations of albumin and TIBC tended to be lower in African Americans across virtually all ages groups, whereas serum creatinine exhibited the opposite trend. Figures 1 through 3 show the distribution of several relevant MBD measures. Among the minerals, serum phosphorus levels appeared essentially the same across the races, whereas serum calcium concentrations were higher in African Americans older than 45 years (Fig. 1). Higher PTH levels in African Americans were persistent regardless of age, whereas serum alkaline phosphatase levels were not different (Fig. 2). The proportion of hemodialysis patients who received an active vitamin D and the average weekly dose among those who received paricalcitol (comprising 94% of administered active vitamin D) was higher in African Americans (Fig. 3).

**Table 1:** Comparing Relevant Demographic, Clinical, and Biochemical Characteristics in the Base Calendar Quarter in 139,328 Maintenance Hemodialysis Patients Including 43,974 African Americans (32%) and 95,354 Non-African Americans (68%)

Age groups (years)		<25	25 to <35	35 to <45	45 to <55	55 to <65	65 to <75	75 to <85	>85
	
	AA	*N* = 668	*N* = 2632	*N* = 5681	*N* = 9560	*N* = 10,479	*N* = 9053	*N* = 4908	*N* = 993
	
	Non-AA	*N* = 1306	*N* = 3733	*N* = 7474	*N* = 13,897	*N* = 20,421	*N* = 23,871	*N* = 20,125	*N* = 4,527
Age (years)	AA	21.5 ± 2.3	30.8 ± 2.7	40.5 ± 2.8	50.3 ± 2.8	60.0 ± 2.8	69.6 ± 2.8	79.0 ± 2.7	88.2 ± 2.6
	Non-AA	21.6 ± 2.2	30.6 ± 2.8	40.5 ± 2.8	50.4 ± 2.8	60.1 ± 2.8	70.1 ± 2.8	79.4 ± 2.7	88.0 ± 2.5
Gender (% female)	AA	46	42	40	41	49	56	60	64
	Non-AA	41	42	38	40	44	45	44	45
Diabetes mellitus (%)	AA	9	19	24	38	51	54	48	38
	Non-AA	5	21	33	46	55	51	39	26
Ethnicity (% Hispanic)	AA	<1	<1	<1	<1	<1	<1	<1	<1
	Non-AA	36	30	26	26	23	19	12	8
Dialysis vintage (%)									
< 6 Months	AA	12	12	11	12	13	14	19	25
	Non-AA	18	15	15	16	17	19	24	29
> 5 Years	AA	24	33	34	32	27	26	20	13
	Non-AA	19	27	26	22	18	16	13	8
Primary insurance (%)									
Medicare	AA	52	59	58	58	58	75	76	78
	Non-AA	45	49	52	50	50	70	73	77
Medicaid	AA	16	11	10	8	7	1	<1	<1
	Non-AA	16	12	9	8	8	2	<1	<1
Marital status (%)									
Married	AA	3	17	23	30	35	33	27	17
	Non-AA	7	26	37	44	49	49	44	32
Divorced	AA	<1	2	6	9	11	8	5	3
	Non-AA	<1	4	8	9	9	6	3	2
Single	AA	79	61	52	41	29	20	15	13
	Non-AA	71	49	36	26	16	10	8	7
Widowed	AA	<1	<1	<1	3	9	20	33	48
	Non-AA	<1	<1	<1	2	7	15	26	39
BMI (kg/m^2^)	AA	26.0 ± 8.4	28.1 ± 8.6	28.4 ± 8.7	28.5 ± 7.9	28.3 ± 7.6	27.1 ± 6.5	25.0 ± 5.7	23.1 ± 6.0
	Non-AA	23.5 ± 6.9	25.5 ± 6.5	26.9 ± 7.6	28.2 ± 7.5	28.2 ± 7.3	26.8 ± 6.4	24.9 ± 5.1	23.5 ± 4.5
*Kt*/*V* (dialysis dose)	AA	1.4 ± 0.4	1.4 ± 0.4	1.4 ± 0.3	1.4 ± 0.3	1.5 ± 0.3	1.5 ± 0.3	1.5 ± 0.3	1.6 ± 0.3
	Non-AA	1.6 ± 0.4	1.5 ± 0.4	1.5 ± 0.4	1.5 ± 0.4	1.5 ± 0.4	1.6 ± 0.4	1.6 ± 0.4	1.6 ± 0.4
Protein catabolic rate	AA	0.9 ± 0.3	0.9 ± 0.3	0.9 ± 0.3	0.9 ± 0.3	0.9 ± 0.2	0.9 ± 0.2	0.9 ± 0.2	0.9 ± 0.2
(g/kg/day)	Non-AA	1.0 ± 0.26	1.01 ± 0.3	1.0 ± 0.3	1.0 ± 0.3	1.0 ± 0.3	1.0 ± 0.3	0.9 ± 0.3	0.9 ± 0.2
Biochemical measures									
Albumin (g/dL)	AA	3.8 ± 0.6	3.8 ± 0.6	3.8 ± 0.5	3.7 ± 0.5	3.7 ± 0.5	3.6 ± 0.5	3.6 ± 0.5	3.4 ± 0.5
	Non-AA	3.9± 0.5	3.9 ± 0.5	3.8 ± 0.5	3.7 ± 0.5	3.7 ± 0.5	3.6 ± 0.5	3.6 ± 0.5	3.5 ± 0.4
Creatinine (mg/dL)	AA	11.8 ± 4.3	11.9 ± 4.3	11.2 ± 3.9	10.1 ± 3.5	8.9 ± 3.1	8.1 ± 2.8	7.3 ± 2.5	6.4 ± 2.3
	Non-AA	10.8 ± 3.5	10.3 ± 3.8	9.3 ± 3.5	8.2 ± 3.1	7.3 ± 2.8	6.7 ± 2.5	6.1 ± 2.3	5.6 ± 2.0
TIBC (mg/dL)	AA	195 ± 41	198 ± 42	207 ± 44	211 ± 46	204 ± 46	196 ± 43	188 ± 43	179 ± 44
	Non-AA	208 ± 43	211 ± 44	212 ± 46	215 ± 48	214 ± 47	210 ± 47	205 ± 46	200 ± 46
Phosphorus (mg/dL)	AA	6.6 ± 1.7	6.3 ± 1.7	6.1 ± 1.6	5.8 ± 1.5	5.5 ± 1.4	5.1 ± 1.3	4.9 ± 1.3	4.6 ± 1.3
	Non-AA	6.7 ± 1.8	6.5 ± 1.8	6.3 ± 1.7	6.0 ± 1.6	5.6 ± 1.5	5.3 ± 1.4	5.0 ± 1.3	4.8 ± 1.2
Calcium (mg/dL)	AA	9.2 ± 0.9	9.2 ± 0.9	9.2 ± 0.8	9.3 ± 0.8	9.3 ± 0.7	9.4 ± 0.7	9.3 ± 0.7	9.2 ± 0.7
	Non-AA	9.2 ± 0.9	9.2 ± 0.8	9.2 ± 0.8	9.2 ± 0.8	9.2 ± 0.7	9.2 ± 0.7	9.2 ± 0.7	9.1 ± 0.7
Intact PTH (ng/mL)	AA	671 ± 604	611 ± 563	576 ± 518	514 ± 477	449 ± 408	400 ± 347	372 ± 331	342 ± 301
	Non-AA	575 ± 588	501 ± 497	438 ± 416	375 ± 357	326 ± 306	292 ± 265	268 ± 235	265 ± 213
Alkaline	AA	144 ± 121	130 ± 98	137 ± 115	141 ± 118	132 ± 96	121 ± 77	116± 72	110 ± 75
phosphatase (U/L)									
	Non-AA	141 ± 164	132 ± 100	136 ± 107	138 ± 110	133 ± 88	122 ± 81	114 ± 66	113 ± 59
Ferritin (ng/mL)	AA	477 ± 486	539 ± 654	529 ± 561	538 ± 507	576 ± 547	586 ± 518	598 ± 555	584 ± 488
	Non-AA	399 ± 455	434 ± 455	455 ± 452	492 ± 502	501 ± 479	521 ± 486	512 ± 476	491 ± 428
Hemoglobin (g/dL)	AA	11.5 ± 1.8	11.6 ± 1.6	11.7 ± 1.5	11.8 ± 1.5	11.8 ± 1.4	11.8 ± 1.4	11.9 ± 1.4	11.8 ± 1.4
	Non-AA	11.9 ± 1.5	11.9 ± 1.5	11.9 ± 1.5	11.9 ± 1.4	12.0 ± 1.4	12.0 ± 1.3	12.0 ± 1.3	12.1 ± 1.3
White blood count (× 10^3^/L)	AA	7.1 ± 2.6	6.8 ± 2.4	6.8 ± 2.3	6.9 ± 2.3	7.0 ± 2.4	6.9 ± 2.4	6.9 ± 2.7	6.9 ± 2.6
	Non-AA	7.2 ± 2.4	7.5 ± 2.4	7.6 ± 2.6	7.7 ± 2.5	7.7 ± 2.7	7.7 ± 2.6	7.8 ± 2.9	7.9 ± 3.3
Lymphocyte	AA	26 ± 10	25 ± 9	24 ± 8	23 ± 8	23 ± 8	22 ± 8	22 ± 8	21 ± 8
	Non-AA	24 ± 9	23 ± 8	20 ± 7	20 ± 7	19 ± 7	18 ± 7	18 ± 7	18 ± 7
(% of total white blood cell count)									
% active vitamin D^a^	AA	62	63	65	67	66	69	67	67
	Non-AA	46	49	51	51	51	51	50	53
Paricalcitol dose^b^ (µg/ week)	AA	20.4 ± 12.3	20.1 ± 11.1	20.0 ± 10.9	18.8 ± 10.1	17.6 ± 9.5	16.0 ± 8.9	14.7 ± 8.4	13.6 ± 7.7
	Non-AA	15.7 ± 10.5	15.3 ± 9.2	15.1 ± 9.2	14.2 ± 9.2	13.2 ± 8.2	12.0 ± 7.4	10.9 ± 6.5	10.3 ± 6.0

*Note:* Values are in percentage or mean ± SD, as appropriate.

a Active vitamin D compounds included paricalcitol, calcitriol, and doxercalciferol.

b Among those who had received any paricalcitol dose during each of the 20 calendar quarters.

**Fig. 4:**
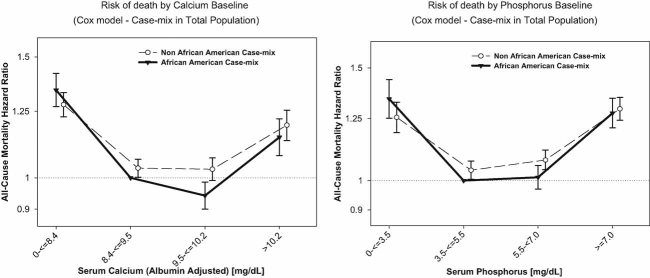
Death hazard ratios (and 95% confidence intervals) of 5-year average corrected albumin-adjusted serum calcium levels in 139,328 hemodialysis patients including 43,974 African Americans (32%) and 95,354 non-African Americans (68%) from July 2001 to June 2001 across four a priori selected increments of serum calcium (*left panel*) and phosphorus (*right panel*) concentrations. Reference group in each analysis is African-American hemodialysis patient population with a KDOQI-recommended target range, that is, calcium level 8.4 to 9.5 mg/dL and phosphorus level 3.5 to 5.5 mg/dL, respectively.

**Fig. 5:**
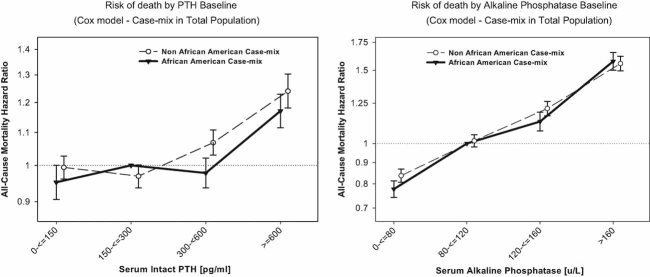
Death hazard ratios (and 95% confidence intervals) of 5-year average corrected albumin-adjusted serum calcium levels in 139,328 hemodialysis patients including 43,974 African Americans (32%) and 95,354 non-African Americans (68%) from July 2001 to June 2001 across four a priori selected increments of serum intact PTH (*left panel*) and alkaline phosphatase (*right panel*) concentrations. Reference group in each analysis is African-American hemodialysis patient population with a recommended target range, that is PTH 150 to 300 pg/mL and alkaline phosphatase 80 to 120 U/L, respectively.

Figures 4 and 5 show the comparison of the case-mix-adjusted mortality predictability of the MBD surrogates in all (incident and prevalent combined) African Americans versus non-African-American hemodialysis patients. Most selected cutoff levels were consistent with the Kidney Disease Outcome Quality Initiative (KDOQI) recommended target values,(30) and the reference group is the target range in African Americans. In African American but no other patients, a serum calcium concentration between 9.5 and 10.2 mg/dL was associated with the greatest survival, whereas both hypocalcemia (<8.4 mg/dL) and hypercalcemia (>10.2 mg/dL) were associated with increased death risk (Fig. 4, *left panel*). Similarly, increased mortality was observed with extreme levels of serum phosphorus (Fig. 4, *right panel*). As shown in Fig. 5 (*left panel*), distinct differences across the racial groups were noticed for certain PTH levels in that in non-African-American patients, a serum PTH above 300 pg/mL was associated with substantial death risk (compared with the KDOQI recommended range of 150 to 300 pg/mL), whereas for both races, a PTH above 600 pg/mL was associated with a substantial increase in death risk. Serum alkaline phosphatase showed a rather linear and incremental association with death with virtually no difference across the races (Fig. 5, *right panel*). The same survival analyses but only in 24,509 incident patients showed slightly different associations (see Supplemental Figs. S1 and S2 for comparison).

We identified 49,674 hemodialysis patients (including 19,918 African Americans) who always received some dose of paricalcitol (>0 µg/week) during each of the 20 calendar quarters, whereas 18,981 never received any active vitamin D dose during the cohort. The former group then was divided into those who received up to an average dose of 10 µg/week (*n* = 17,347) and those who received higher doses of paricalcitol (*n* = 32,327). Logistic regression was used to examine factors that are associated with the probability of receiving the highest paricalcitol dose (>10 µg/week) versus the lowest dose (>0 to <10 µg/week) among those who received this medication. As shown in Table 2, African Americans had twice the odds of receiving a higher paricalcitol dose than other races. We note, however, that because the outcome (high dose) is so common, this doubling of the odds is much larger than the increase in probability of a high dose. Higher serum levels of calcium, phosphorus, PTH, or alkaline phosphatase levels were associated with the higher paricalcitol dose.

**Table 2:** Odds Ratio of Receiving the Highest Paricalcitol Dose (≥10 µg/Week, *n* = 32,327) Versus the Lowest Paricalcitol Dose (>0 to <10 µg/Week, *n* = 17,347) in 49,674 Hemodialysis Patients Who Received Any Dose of Paricalcitol During All 20 Calendar Quarters of the Cohort (July 2001 to June 2006)

	Unadjusted	Case-mix adjusted
Age (each 10-year increase)	0.8 (0.7–0.8)*	0.8 (0.8–0.9)*
Gender (women versus men)	1.0 (1.0–1.1)	1.0 (1.0–1.1)
Diabetes mellitus (versus no diabetes)	0.9 (0.8–0.9)*	0.9 (0.9–1.0)*
Race/ethnicity		
Non-Hispanic whites (reference)	1	1
African Americans	2.6 (2.5–2.7)*	2.1 (2.0–2.2)*
Hispanics	1.1 (1.0–1.1)*	0.9 (0.8–1.0)*
Asians	0.7 (0.7–0.8)*	0.6 (0.6–0.7)*
Dialysis treatment vintage time		
< 6 Months (reference)	1	1
6–24 Months	1.1 (1.1–1.2)*	1.0 (1.0–1.1)
2–5 Years	1.7 (1.6–1.8)*	1.5 (1.4–1.6)*
> 5 Years	2.6 (2.4–2.7)*	2.1 (1.9–2.3)*
Primary insurance		
Medicare (reference)	1	1
Medicaid	1.3 (1.1–1.4)*	0.9 (0.9–1.0)*
Private insurance	1.1 (1.0–1.1)	0.9 (0.9–1.1)
*Kt*/*V* (each 0.1-unit increase)	1.0 (0.9–1.0)*	1.0 (0.9–1.0)*
nPCR (nPNA) (each 0.1 g/kg/day increase)	1.0 (1.0–1.1)*	1.0 (1.0–1.1)*
BMI (each 1 kg/m^2^ increase)	1.0 (1.0–1.1)*	1.0 (1.0–1.1)*
Biochemical measures		
Albumin (each 0.1 g/dL increase)	1.1 (1.0–1.1)*	1.0 (1.0–1.1)*
Creatinine (each 1 mg/dL increase)	1.2 (1.1–1.2)*	1.1 (1.0–1.1)*
TIBC (each 10 mg/dL increase)	1.0 (1.0–1.1)*	1.0 (1.0–1.1)*
Phosphorus (each 1 mg/dL increase)	1.4 (1.3–1.4)*	1.3 (1.3–1.4)*
Calcium^a^ (each 1 mg/dL increase)	1.4 (1.3–1.4)*	1.4 (1.3–1.4)*
Ferritin (each 100 ng/mL increase)	1.0 (1.0–1.1)*	1.0 (0.9–1.0)
Hemoglobin (each 1 g/dL increase)	1.0 (0.9–1.0)*	1.0 (0.9–1.0)*
WBC (each 10^3^/µL increase)	0.9 (0.9–1.0)*	1.0 (0.9–1.0)*
Lymphocyte (each 1% of total WBC increase)	1.0 (1.0–1.1)*	1.0 (1.0–1.1)*
Intact PTH (each 100 pg/mL increase)	1.4 (1.3–1.4)*	1.3 (1.3–1.4)*
Alkaline phosphatase (each 10 U/L increase)	1.0 (1.0–1.1)*	1.1 (1.0–1.1)*

nPCR, normalized protein catabolic rate; nPNA, normalized protein nitrogen appearance.

a Serum calcium is adjusted for serum albumin.

* *p* < .001.

**Fig. 6:**
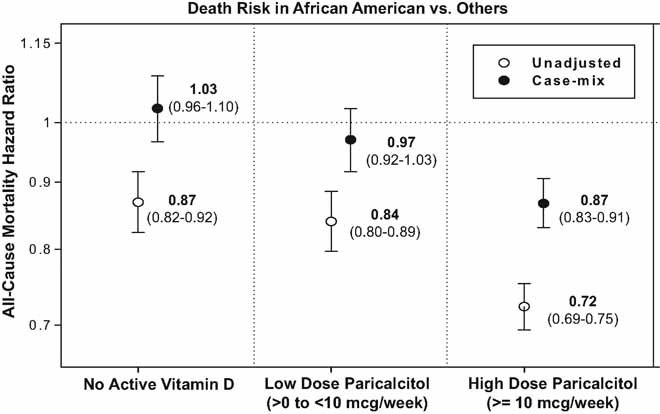
Death hazard ratios of African-American (AA) versus non-AA patients across three mutually exclusive strata of no active vitamin D (*n* = 18,981, *right section*), low paricalcitol dose (>0 and <10 µg/week, *n* = 17,347, *middle section*) and high paricalcitol dose (≥10 µg/week, *n* = 32,327, *left section*). Survival analyses were performed in unadjusted and case-mix-adjusted formats (see text for list of covariates).

Figure 6 shows hazard ratios for death of African Americans compared with non-African Americans across low and high doses of paricalcitol. In the no active vitamin D group and the low-paricalcitol-dose group, African Americans did not show any survival advantages compared with others, whereas among those who received a high paricalcitol dose, 12% greater survival was noticed (Fig. 6). This association was consistent among incident patients (data no shown) and across most age groups (see Supplemental Fig. S3). The association of survival superiority of African Americans and higher paricalcitol dose appeared independent of additional adjustments in sensitivity analyses (see Supplemental Fig. S4 for sensitivity analyses).

## Discussion

Among 139,328 thrice-weekly-treated hemodialysis patients, including 32% African Americans, in a single large dialysis organization, where most laboratory values were measured monthly up to 60 months (July 2001 to June 2006), we found that African-American hemodialysis patients had higher serum calcium and PTH levels than—but similar phosphorus and alkaline phosphatase levels as—other races, implying that the chemical hyperparathyroidism in African Americans may not cause a more severe bone disease. They also were more likely to receive injectable active vitamin D compounds, and when they did, they tended to receive higher doses of these medications. Survival analyses comparing the two races showed subtle but distinct differences in mortality predictabilities of different ranges of serum calcium, phosphorus, and PTH but not alkaline phosphatase. African Americans who received the highest paricalcitol dose (≥10 µg/week) had a demonstrable survival advantage compared with African Americans who received no active vitamin D or only low doses of paricalcitol (<10 µg/week).

These findings may have important implications for the management of dialysis patients of different races, especially since guidelines and conditions for pay for performance might not devote adequate attention to the role of race in the management of CKD-MBD in hemodialysis patients. ESRD incidence and prevalence have been rising in the United States and in most countries around the world.(31) There are currently approximately 400,000 Americans who undergo maintenance dialysis treatment to survive, and one-third of them are African Americans. Racial discrepancies in CKD have persisted over the past 20 years.(3) The annual ESRD incidence for African Americans in 2006 reached 1010 per million, which is 3.6 times greater than non-Hispanic whites.(31) Prevalence of ESRD continues to be highest for African Americans, at 5004 per million population in 2006, compared with 1194 among whites.(31) In many dialysis clinics in inner-city areas, over half to two-thirds of dialysis patients are African American.(31) The median age of the prevalent ESRD population, which is 58.8 years, ranges from 56.9 years in the African Americans to 60.0 years among whites.(6,31) As shown in Table 1, in our study across virtually all age groups, approximately one-third of the dialysis patients were African Americans compared with 14% of the US general population.(31)

Among diverse CKD complications, MBDs are more prevalent in African Americans, in whom PTH levels are elevated in comparison with other races.(11–15) In a recent descriptive cohort study in 1860 predialysis CKD patients including 227 African Americans, the latter patients had lower levels of 25-hydroxyvitamin D [25(OH)D, calcidiol], almost the same levels of 1,25-dihydroxyvitamin D_3_ [1.25(OH)_2_D_3_, calcitriol] but higher levels of calcium, phosphorus, and PTH, and were more likely to have hyperphosphatemia than other races.(32) In our prevalent hemodialysis cohort we found that serum calcium and PTH levels were higher in African Americans, but serum phosphorus and alkaline phosphatase levels were not (Figs. 1 and 2), even though these patients were more likely to have received active vitamin D and at even higher doses, which should increase gastrointestinal phosphorus absorption. These unique features of CKD-MBD in the African-American race suggest that more complicated mechanisms exist that deserve further investigations based on novel hypotheses.

In the general population without overt CKD, although African Americans have lower levels of circulating calcidiol, there is no increased musculoskeletal disease that would be expected owing to such a deficiency, and African Americans indeed have lower rates of osteoporotic fractures.(33) There are several theories about the protective mechanisms of vitamin D deficiency. A potential adaptive response may involve compensatory increases in functionality of circulating calcitriol, that is, active vitamin D, that depends on 1α-hydroxylase in the kidneys and which is tightly regulated by circulating levels of calcium, phosphorus, and PTH. With moderately low levels of 25(OH)_2_D_3_, there is upregulation of PTH with subsequently increased renal 1,25(OH)_2_D_3_ production.(34)

There is also evidence that in African Americans, intestinal vitamin D receptors are less susceptible to the mineral absorptive actions of 1,25(OH)_2_D_3_. The foregoing combination may lead to the beneficial bone effects of increased 1,25(OH)_2_D_3_ without hyperphosphatemia or only mild hypercalcemia in African Americans, as we have observed in our national database.(34) Hence it is possible that active vitamin D supplementation is uniquely crucial in African-American CKD patients, in whom the foregoing protective effects wane with progressively less renal 1α-hydroxylation.

Although dialysis therapy is expected to be lifesaving, ironically, approximately 1 of every 5 American dialysis patients dies each year, that is a 5-year survival of only approximately 35%,(5,35) which is worse than most cancers in the twenty-first century.(5,35,36) At any given age, group dialysis mortality is 10 to 100 times higher than that of nondialysis Medicare patients.(4,31,37) Almost half the deaths are attributed to cardiovascular disease.(35,38,39) Yet, for reasons that are as yet unknown, African-American dialysis patients have greater survival than their non-Hispanic white counterparts, a finding that has persisted over the past two decades.(4,5) The greater survival of African Americans is robust and consistent not only irrespective of demographic or residency status but also independent of the type of dialysis (hemodialysis [HD] versus peritoneal dialysis), dialysis dose, or other factors related to dialysis treatment or technique. The racial disparities persist for cause-specific mortality: For instance, African-American dialysis patients are 17% less likely to die of cardiovascular disease than whites.(31,37)

Recent observational studies have suggested a rather persistent mortality predictability of high serum calcium and phosphorus levels in CKD patients, in particular, in maintenance dialysis patients.(40–42) These associations are often attributed to increased cardiovascular risk as a results of enhanced calcification of the vessels, including coronary arteries, on calcium and phosphorus load according to some(43,44) but not all(45,46) studies. We found that in ranges around the KDOQI-recommended target for calcium and phosphorus, African-American hemodialysis patients had slight greater survival chances than other races (Fig. 4). Additional subtle differences were noticed, including the observation that a borderline high serum calcium concentration of 9.5 to 10.2 mg/dL was associated with the greatest survival in African Americans but not other hemodialysis patients (Fig. 4), although in incident patients, some of these associations appeared different. Moderately high PTH levels exhibited slightly different mortality predictabilities in moderately high ranges, whereas virtually no difference was observed for alkaline phosphatase (Fig. 5). Alkaline phosphatase has been shown both in previous studies(23,46,47) and in this study to be the only BMD marker with a strictly linear and incremental mortality predictability, whereas other MBD surrogates exhibit rather U-shaped associations (Figs. 4 and 5).

A recent study in 9303 incident hemodialysis patients including 3214 African Americans by Wolf and colleagues(16) suggested that therapy with activate vitamin D may be a potential explanation for racial survival differences in incident hemodialysis patients, especially since African-American CKD patients, by virtue of having higher PTH levels, are more likely to receive injectable vitamin D compounds in dialysis clinics. In our study we found that low paricalcitol doses (<10 µg/week) were not associated with survival differences across races, whereas higher doses (>10 µg/week) were so (Fig. 6). As discussed earlier, there may be inherent constellations of PTH and active vitamin D that are crucial to the musculoskeletal and cardiovascular physiologies of African Americans in general and the subgroup with CKD in particular.

A limitation of our study is that we did not have access to the intake and types of phosphorus binders or other oral medications related to MBDs, such as oral nutritional vitamin D. However, it is highly unlikely that US dialysis patients, especially during the 2001–2006 era, received nutritional vitamin D,(48) even though this practice pattern may be more common in other countries. Although it could be argued that minerals, PTH, and even alkaline phosphatase are significantly affected by calcimimetics, a new group of medications,(29) our cohort belonged to the precalcimimetic era. We also lacked detailed and updated data on comorbid states and explicit laboratory markers of inflammation such as C-reactive protein. However, as discussed earlier, we believe that adjustment for the MICS may be inappropriate and introduce new sources of errors and bias, especially if survival benefits of higher doses of active vitamin D are exerted though modulating the MICS-related pathways, among others. Hence we think that the case-mix-adjusted models are the most appropriate ones.

The strengths of our study include (1) its contemporary nature, since all patient data were obtained from the twenty-first century (2001–2006), (2) its uniform laboratory measurements, with all laboratory data obtained from one single facility, (3) its large sample size, (4) its time-averaged *Kt*/*V* and laboratory data, with most values representing means of up to 3-monthly measurements, and (5) its examination of a 5-year cohort rather than shorter (1-to 3-year) periods of time used in previous studies.(49,50)

## Conclusions

Among 139,328 thrice-weekly-treated hemodialysis patients, including 32% African Americans, in a single large dialysis organization, where most laboratory values were measured monthly for up to 60 months (July 2001 to June 2006), we found that African Americans had higher serum calcium and PTH levels but similar concentrations of phosphorus and alkaline phosphatase and were more likely to receive injectable active vitamin D medications and at higher doses than their non-African-American counterparts. Subtle but distinct differences in mortality predictabilities of different ranges of serum calcium, phosphorus, and PTH but not alkaline phosphatase were observed across the two groups and among incident versus prevalent patients. African Americans who had received higher doses of paricalcitol (>10 µg/week) had a demonstrable survival advantage compared with African Americans who had received no active vitamin D or lower doses. Given both current KDOQI guidelines and upcoming conditions for pay for performance under the imminent bundling system that may lead to changes in the management patterns of CKD-MBD among US dialysis patients, our findings may have important implications and, if verified, may suggest more attention to the inclusion of race as an independent adjustor in the bundling equations.
