# Supplementary material for: Impact of Race on Hyperparathyroidism, Mineral Disarrays, Administered Vitamin D Mimetic, and Survival in Hemodialysis Patients
Source: J Bone Miner Res. 2010 Jul 7;25(12):2724–34. doi: 10.1002/jbmr.177 (PMC3179282; doi:10.1002/jbmr.177)

On-Line Only Material - Electronic Appendix

Table A.1. Comparing relevant demographic, clinical and biochemical characteristics in the base calendar quarter in 12,730 maintenance hemodialysis patients who were not followed beyond 45 days vs. 139,328 maintenance hemodialysis patients including 24,509 incident patients (vintage <6 months) and 114,643 prevalent patients (vintage >=6 months)

Variable		**Not followed >45 days**	**Vintage <6 mos (incident patients)**	**Vintage >=6 mos (prevalent patients)**
	AA	**n= 2,481**	**n= 6,063**	**n= 37,888**
	non AA	**n= 10.249**	**n= 18,446**	**n= 76,755**
Age	AA	61.4 ± 17.0	60.3 ± 15.6	57.4 ± 14.8
	non AA	64.9 ± 19.3	66 ± 15.3	62.8 ± 15.3
Gender (% female)	AA	50	49	49
	non AA	42	43	43
Diabetes mellitus (%)	AA	42	47	42
	non AA	33	45	44
Ethnicity (% Hispanic)	AA	0	0	0
	non AA	14	16	21
**Primary insurance (%)**				
Medicare	AA	47	53	66
	non AA	45	56	62
Medicaid	AA	8	9	5
	non AA	5	6	5
Private Insurance	AA	7	6	9
	non AA	9	7	11
Other	AA	33	26	11
	non AA	33	24	13
**Marital Status (%)**				
Married	AA	25	29	29
	non AA	37	45	44
Divorced	AA	6	7	8
	non AA	4	6	6
Single	AA	28	35	33
	non AA	14	16	18
Widowed	AA	14	14	11
	non AA	14	15	12
BMI	AA	27.2 ± 8.4	27.1 ± 7.7	27.7 ± 7.6
	non AA	26.7 ± 8.8	26.5 ± 7.3	26.7 ± 6.7
Kt/V (dialysis dose)	AA	1.4 ± 0.5	1.4 ± 0.4	1.5 ± 0.3

	non AA	1.4 ± 0.5	1.5 ± 0.4	1.6 ± 0.4
Protein Catabolic Rate (g/kg/day)	AA	0.8 ± 0.3	0.8 ± 0.3	0.9 ± 0.2
	non AA	0.8 ± 0.3	0.9 ± 0.3	1 ± 0.3
**Serum levels**				
albumin (g/dL)	AA	3.1 ± 0.6	3.4 ± 0.6	3.7 ± 0.4
	non AA	3.3 ± 0.6	3.4 ± 0.6	3.7 ± 0.4
creatinine (mg/dL)	AA	6.2 ± 3.2	7.1 ± 3.1	9.7 ± 3.5
	non AA	5.2 ± 2.5	5.8 ± 2.6	7.7 ± 3
TIBC (mg/dL)	AA	192 ± 58	198 ± 54	202 ± 43
	non AA	206 ± 63	207 ± 54	211 ± 45
phosphorus (mg/dL)	AA	4.8 ± 1.7	5.1 ± 1.5	5.6 ± 1.5
	non AA	4.8 ± 1.6	5.2 ± 1.5	5.6 ± 1.5
Calcium (mg/dL)	AA	8.9 ± 0.8	9.1 ± 0.8	9.3 ± 0.8
	non AA	8.8 ± 0.8	9 ± 0.7	9.2 ± 0.7
Intact PTH (ng/mL)	AA	439 ± 422	392 ± 382	461 ± 446
	non AA	342 ± 350	282 ± 279	313 ± 327
Alkaline phos. (U/L)	AA	157 ± 177	132 ± 120	121 ± 94
	non AA	136 ± 120	129 ± 110	117 ± 82
Ferritin (ng/mL)	AA	688 ± 948	515 ± 651	570 ± 520
	non AA	494 ± 673	438 ± 539	515 ± 462
Blood hemoglobin (g/dL)	AA	10.3 ± 1.5	11.7 ± 1.7	11.9 ± 1.4
	non AA	10.7 ± 1.5	11.9 ± 1.6	12.1 ± 1.3
WBC ($x10^3$/υl)	AA	9.5 ± 4.6	7.7 ± 3.1	6.8 ± 2.2
	non AA	9.6 ± 4.5	8.4 ± 3.5	7.6 ± 2.4
Lymphocyte (% of total WBC)	AA	17 ± 8	21 ± 9	23 ± 8
	non AA	15 ± 8	18 ± 8	19 ± 7
% active vitamin D*	AA		51	69
	non AA		43	53
Paricalcitol dose** (mcg/week)	AA		15.5 ± 9	17.8 ± 9.9
	non AA		11.9 ± 7.5	12.8 ± 8.1

Baseline values for African American (AA) versus non African American (non AA). Non African American includes all other races.

On-Line Figures

On-Line Figure Legends

Figure A.1. Death hazard ratios (and 95% confidence intervals) of five-year averaged corrected albumin adjusted serum calcium levels in 24,509 hemodialysis patients including 6,063 African Americans (25%) and 18,446 non-African Americans (75%) from 7/2001 to 6/2001 across 4 *a priori* selected increments of serum calcium (left panel) and phosphorus (right panel) concentrations. Reference group in each analysis is African American hemodialysis patient population with a KDOQI recommended target range, i.e., calcium 8.4 to 9.5 mg/dL and phosphorus 3.5 to 5.5 mg/dL, respectively.

Figure A.2. Death hazard ratios (and 95% confidence intervals) of five-year averaged corrected albumin adjusted serum calcium levels in 24,509 hemodialysis patients including 6,063 African Americans (25%) and 18,446 non-African Americans (75%) from 7/2001 to 6/2001 across 4 *a priori* selected increments of serum intact PTH (left panel) and alkaline phosphatase (right panel) concentrations. Reference group in each analysis is African American hemodialysis patient population with a recommended target range, i.e., PTH 150 to 300 pg/mL and alkaline phosphatase 80 to 120 U/L, respectively.

Figure A.3. Death hazard ratios of African Americans (AA) vs. non-African-American hemodialysis patients according to paricalcitol dose among those who had received this medication across eight 10-yaer age increments. The paricalcitol dose strata are mutually exclusive (>0 and <10 mcg/wk, n=17,347, vs. >=10 mcg/wk, n=32,327). Survival analyses are performed in

unadjusted and case-mix adjusted formats (see text for list of covariates). Note that in the 2 marginal groups (<25 years and >85 years old) there was not enough subjects for low dose analyses.

Figure A.4. Sensitivity analyses to examine the robustness of the survival advantage of AA vs. non-AA patients in case-mix survival models after inclusion of additional adjustors in the Cox regression model.

On-Line Figure A.1.

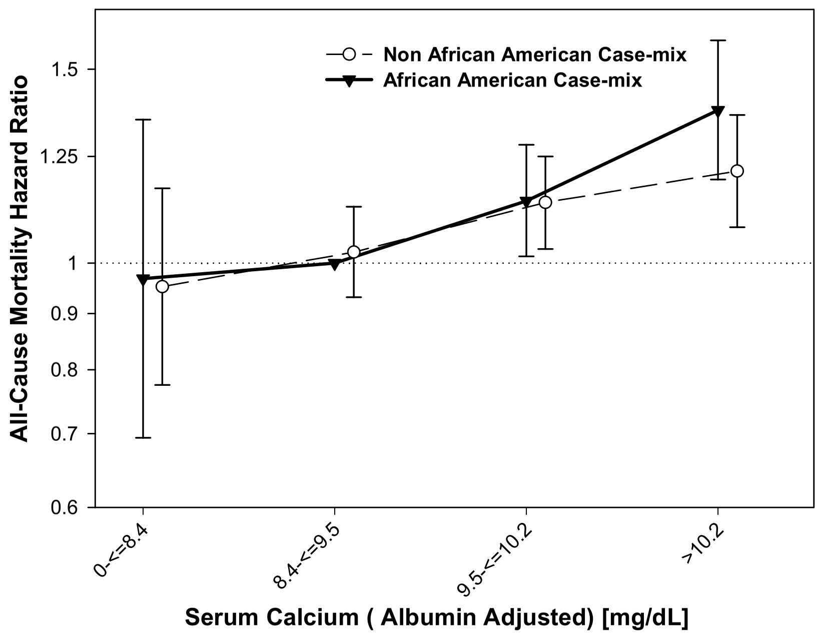

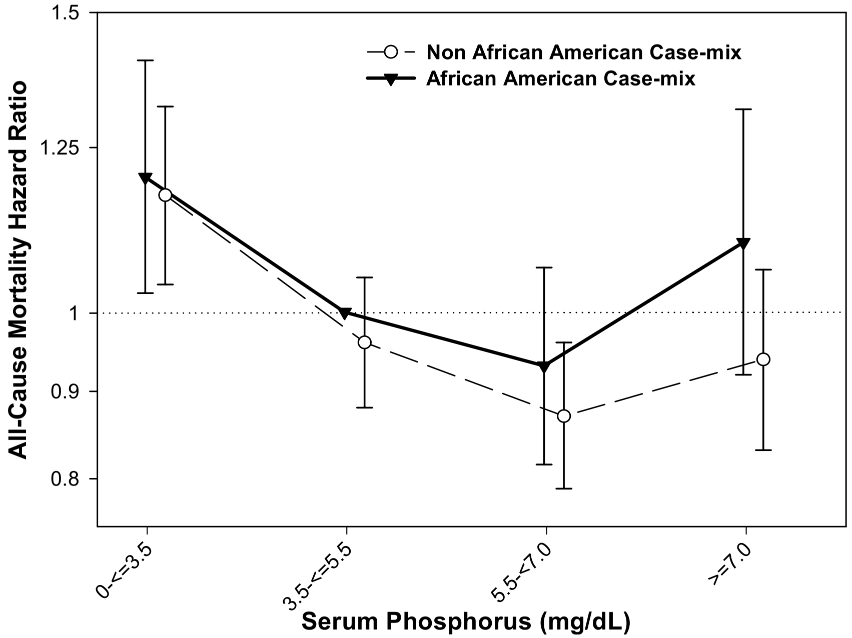

On-Line Figure A.2.

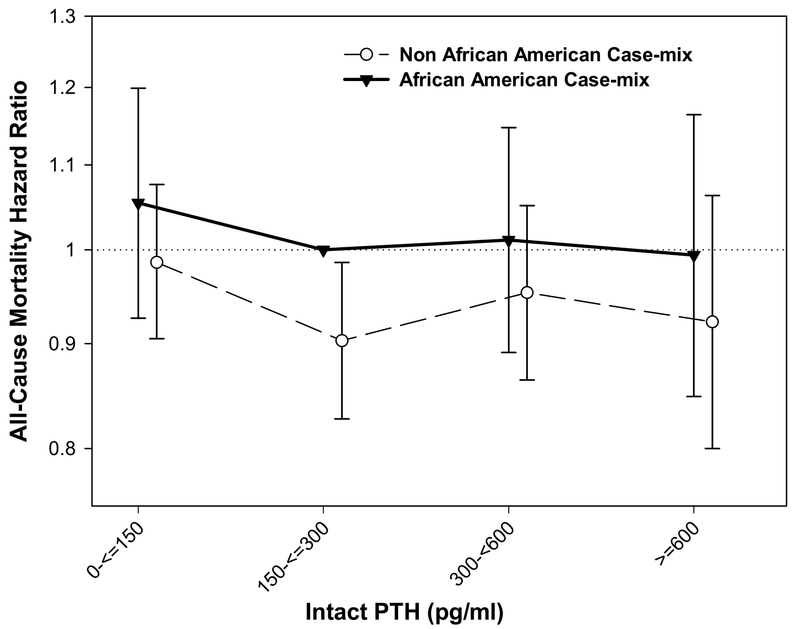

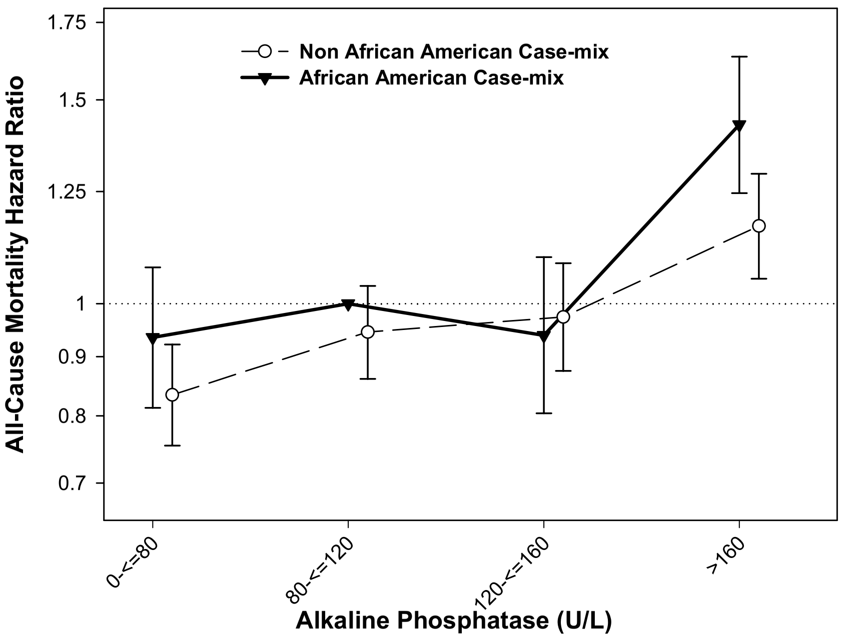

On-Line Figure A.3.

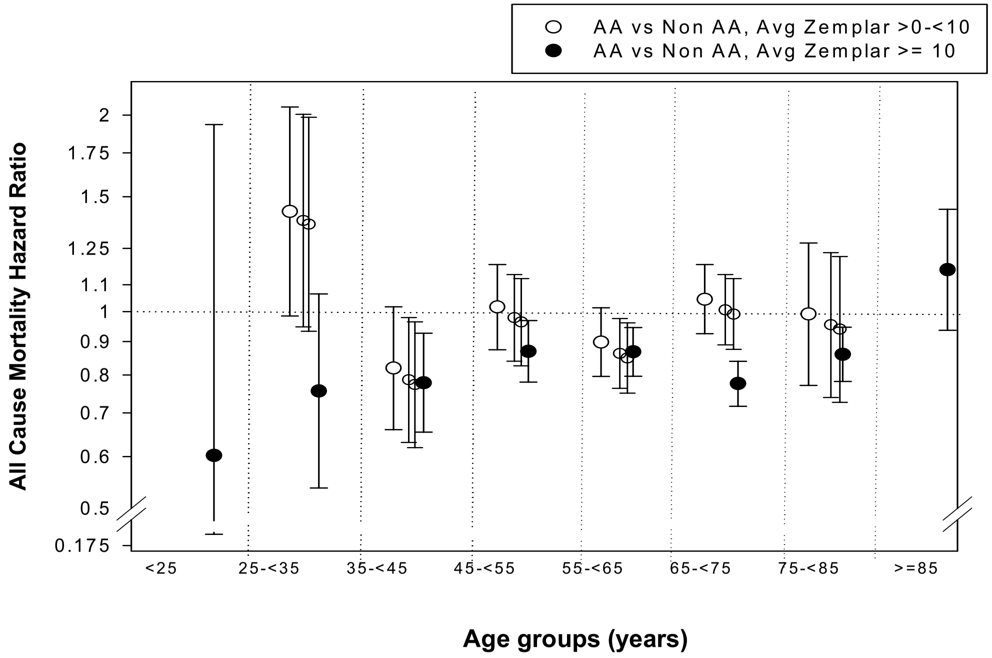

On-Line Figure A.4

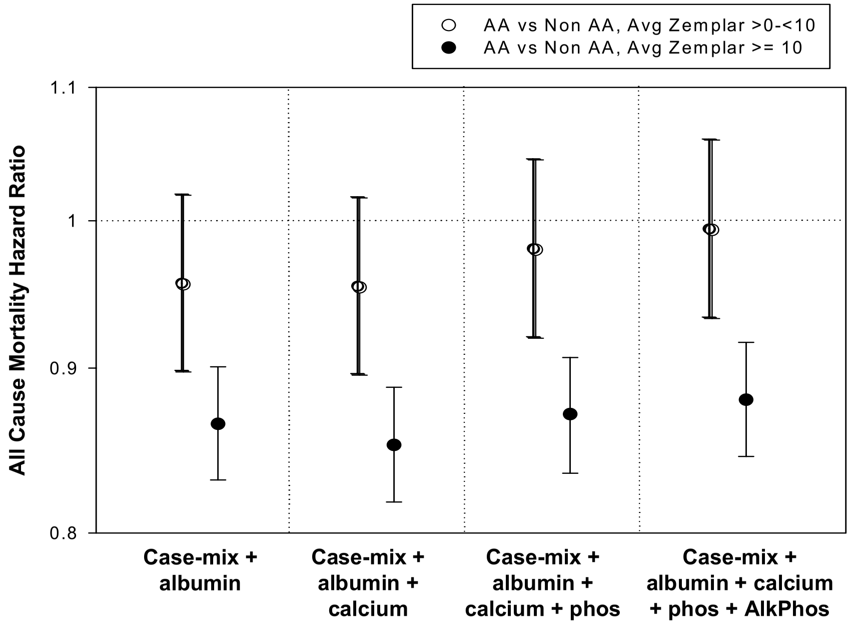

Supplement: Supplementary file 1 [file jbmr0025-2724-SD1.pdf]
